# New mutation in the β1 propeller domain of LRP4 responsible for congenital myasthenic syndrome associated with Cenani–Lenz syndrome

**DOI:** 10.1038/s41598-023-41008-5

**Published:** 2023-08-28

**Authors:** Marion Masingue, Olivia Cattaneo, Nicolas Wolff, Céline Buon, Damien Sternberg, Morgane Euchparmakian, Myriam Boex, Anthony Behin, Kamel Mamchaouhi, Thierry Maisonobe, Marie-Christine Nougues, Arnaud Isapof, Bertrand Fontaine, Julien Messéant, Bruno Eymard, Laure Strochlic, Stéphanie Bauché

**Affiliations:** 1 INSERM, Myology Research Center-UMRS974, Hôpital Universitaire de la Pitié-Salpêtrière, Institut de Myologie, Sorbonne Université, 105 Boulevard de l’Hôpital, 75013 Paris, France; 2 Service de Neuromyologie, Centre de Référence Neuromusculaire, APHP, Paris, France; 3 Department of Cell Physiology and Metabolism, University of Geneva, Geneva, Switzerland; 4 Institut Pasteur, Channel Receptors Unit, UMR CNRS 3571, Université de Paris, Paris, France; 5 Service de Biochimie Métabolique, UF Cardiogenetics and Myogenetics, Hôpital de la Pitié-Salpêtrière, APHP, Paris, France; 6 Département de Neurophysiologie Clinique, Centre de Référence des Pathologies Neuromusculaires, Hôpital de la Pitié-Salpêtrière, APHP, Paris, France; 7 Département de Neuropédiatrie, Centre de Référence des Pathologies Neuromusculaires, Hôpital Trousseau, APHP, Paris, France; 8 Service de Neuromyologie, Centre de Référence Canalopathie, Hôpital de la Pitié-Salpêtrière, APHP, Paris, France

**Keywords:** Developmental biology, Neuroscience, Neurology

## Abstract

Congenital myasthenic syndromes (CMS) are a clinically and genetically heterogeneous group of rare diseases due to mutations in neuromuscular junction (NMJ) protein-coding genes. Until now, many mutations encoding postsynaptic proteins as Agrin, MuSK and LRP4 have been identified as responsible for increasingly complex CMS phenotypes. The majority of mutations identified in *LRP4* gene causes bone diseases including CLS and sclerosteosis-2 and rare cases of CMS with mutations in *LRP4* gene has been described so far. In the French cohort of CMS patients, we identified a novel *LRP4* homozygous missense mutation (c.1820A > G; p.Thy607Cys) within the β1 propeller domain in a patient presenting CMS symptoms, including muscle weakness, fluctuating fatigability and a decrement in compound muscle action potential in spinal accessory nerves, associated with congenital agenesis of the hands and feet and renal malformation. Mechanistic expression studies show a significant decrease of AChR aggregation in cultured patient myotubes, as well as altered in vitro binding of agrin and Wnt11 ligands to the mutated β1 propeller domain of LRP4 explaining the dual phenotype characterized clinically and electoneuromyographically in the patient. These results expand the *LRP4* mutations spectrum associated with a previously undescribed clinical association involving impaired neuromuscular transmission and limb deformities and highlighting the critical role of a yet poorly described domain of LRP4 at the NMJ. This study raises the question of the frequency of this rare neuromuscular form and the future diagnosis and management of these cases.

## Introduction

Mutations affecting a single gene can be associated to various forms of genetic diseases. This is the case for *LRP4* mutations that are responsible for three different pathological phenotypes in humans: CLS (MIM 212780), sclerosteosis 2 (MIM 614305) with or without syndactylies (MIM 185900) and CMS (MIM 616304)^1–5^. The phenotypic heterogeneity of these diseases ranging from congenital malformations affecting the limbs for CLS and sclerosteosis 2 to fatigability and muscle weakness for CMS suggest that LRP4 may have several specific functions acting on different organs in a timely manner. Until now, the majority of mutations identified in the *LRP4* gene causing bone diseases including CLS and sclerosteosis-2 are the result of the role of LRP4 signaling in autopod formation and kidney development^1–3^.

LRP4 also plays a crucial role in the formation and maintenance of the neuromuscular junction (NMJ) orchestrated by the agrin/LRP4/MuSK signaling pathway^6, 7^. Neural agrin released from the nerve terminal of the motor neuron binds to the β1 propeller domain of LRP4 enhancing activation of the tyrosine kinase domain of MuSK associated to LRP4 on its third β propeller domain^8, 9^. This activation aggregates and anchors the AChRs clusters on the muscle membrane under the nerve terminal, and promotes the expression of postsynaptic genes. Multiple mutations in *AGRN* and *MUSK* genes have been identified as responsible for CMS cases while only a few cases of CMS with mutations in the *LRP4* gene have been described so far^5, 10–14^. In addition to this major signaling pathway, several secreted Wnt glycoproteins contribute to LRP4/MuSK signaling regulating NMJ formation and interact directly with the ecto domain of LRP4 in vitro^15^*.*

Using Sanger sequencing, we identified a novel *LRP4* homozygous missense mutation (c.1820A > G; p.Thy607Cys) within the β1 propeller domain in a patient presenting CMS symptoms, including muscle weakness, fluctuating fatigability and a decrement in compound muscle action potential (CMAP) in spinal accessory nerves, associated with congenital agenesis of the hands and feet, and renal malformation. In vitro expression studies revealed the decrease of agrin and Wnt11 binding to mutated β1 propeller domain leading to a disruption of LRP4/MuSK signaling associated with a strong decrease of AChR aggregation in the patient cultured myotubes.

## Results

### Clinical data

**Figure 1:**
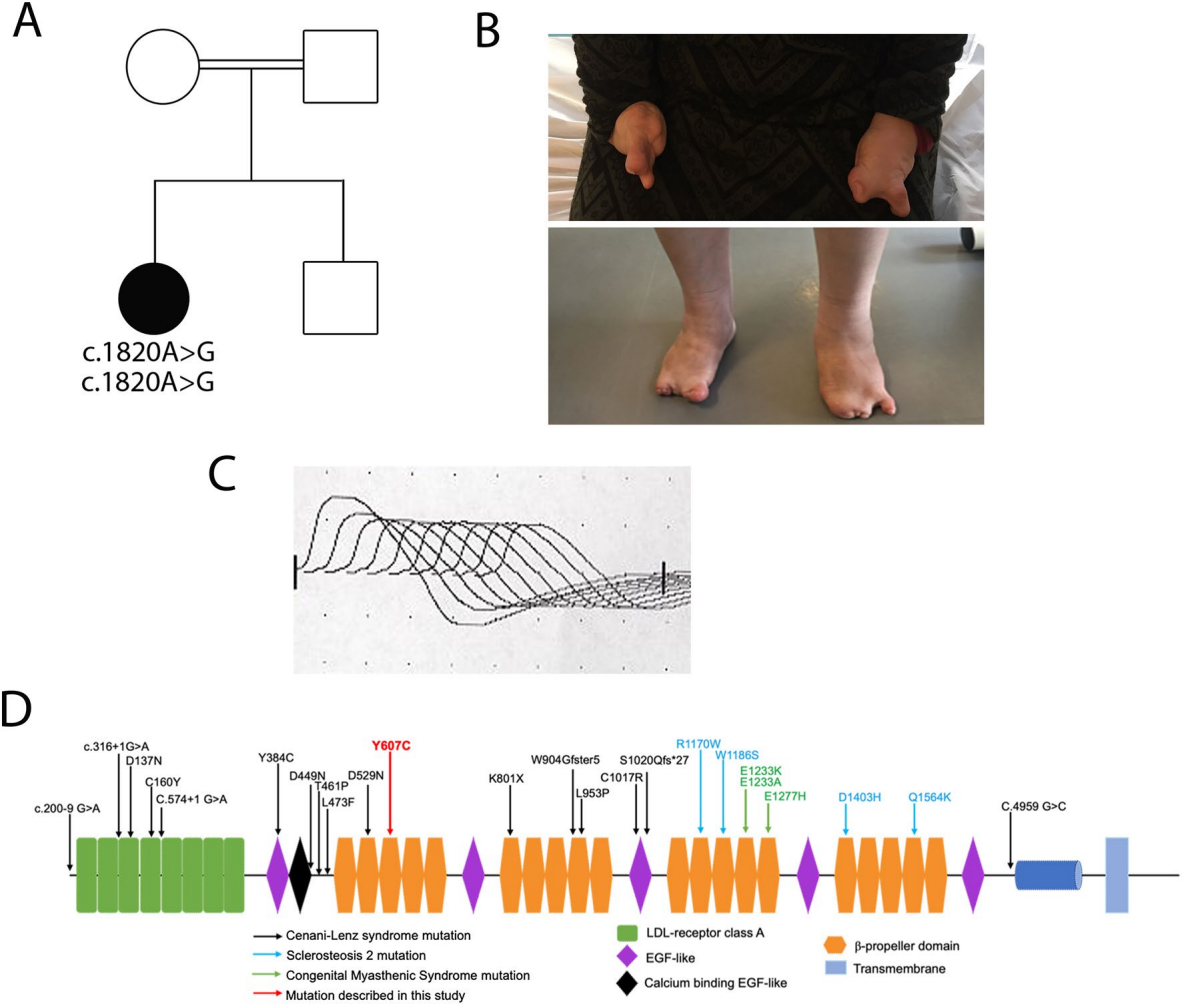
Genetic and electrophysiologic features of recessive *LRP4* mutation. (**A**) The family pedigree from the consanguineous family at first degree revealed that the proband (in black) is affected. (**B**) Image of the patient’s congenital agenesis of the hands and feet. (**C**) Decrement at RNS (3 Hz) was observed from 25 to 28% in spinal accessory nerves. (**D**) Position of the identified mutation on the structure of human LRP4 protein. Missense mutation identified in the β1 propeller domain in this study is indicated in red (Y607C), whereas mutations described in CMS are indicated in green. Mutations described in Cenani Lenz syndrome are represented in black and mutations described in sclerosteosis 2 are represented in blue. For A and B figures were drawn by using BioRender.com.

The patient, a 42 years old Algerian woman, was born from consanguineous parents. The parents and brother are unaffected (Fig. 1A). She was born after normal gestation and delivery, with congenital malformations of the hands and feet characterized by syndactyly and brachydactyly (more severe for the hands), and a renal malformation with a horseshoe-shaped kidney compatible with a CLS (Fig. 1B). At the age of 19, she started experiencing progressively worsening muscle weakness, with cervical fatigability. Upon examination at 42 years old, she presented with a clinical picture of CMS associating proximal muscle weakness in all 4 limbs predominantly on her left hemi-body, as well as cervical and abdominal deficiencies. Transient at first, those symptoms have become permanent since 3 to 4 years, but still worsened with exercise or fatigue. There was no oculomotor disorder and no bulbar signs. To address the possibility of myasthenia gravis, antibodies against AChR and MuSK in the patient’s serum were tested and were negative. RNS at 3 Hz revealed a decremental response of 25–28% in the spinal accessory nerves compared with the first evoked CMAP (normal < 10%; Fig. 1C). Furthermore, single nerve stimulations did not induce repetitive CMAP (data not shown), resulting from the prolongation of synaptic responses, which is observed exclusively in slow channel syndrome and acetylcholinesterase deficiency. Histological analysis of muscle sections revealed fiber size variability on H&E staining and marked type I fiber predominance on ATPase staining which is a usual feature in CMS even if not specific (Supplemental Fig. 2), without detection of NMJ on the patient's biopsy. Anticholinesterase treatment did not improve the CMS symptoms and the efficiency of salbutamol treatment, in the absence of response to anticholinesterase treatment, could not be evaluated because the patient has been lost from sight.

**Figure 2:**
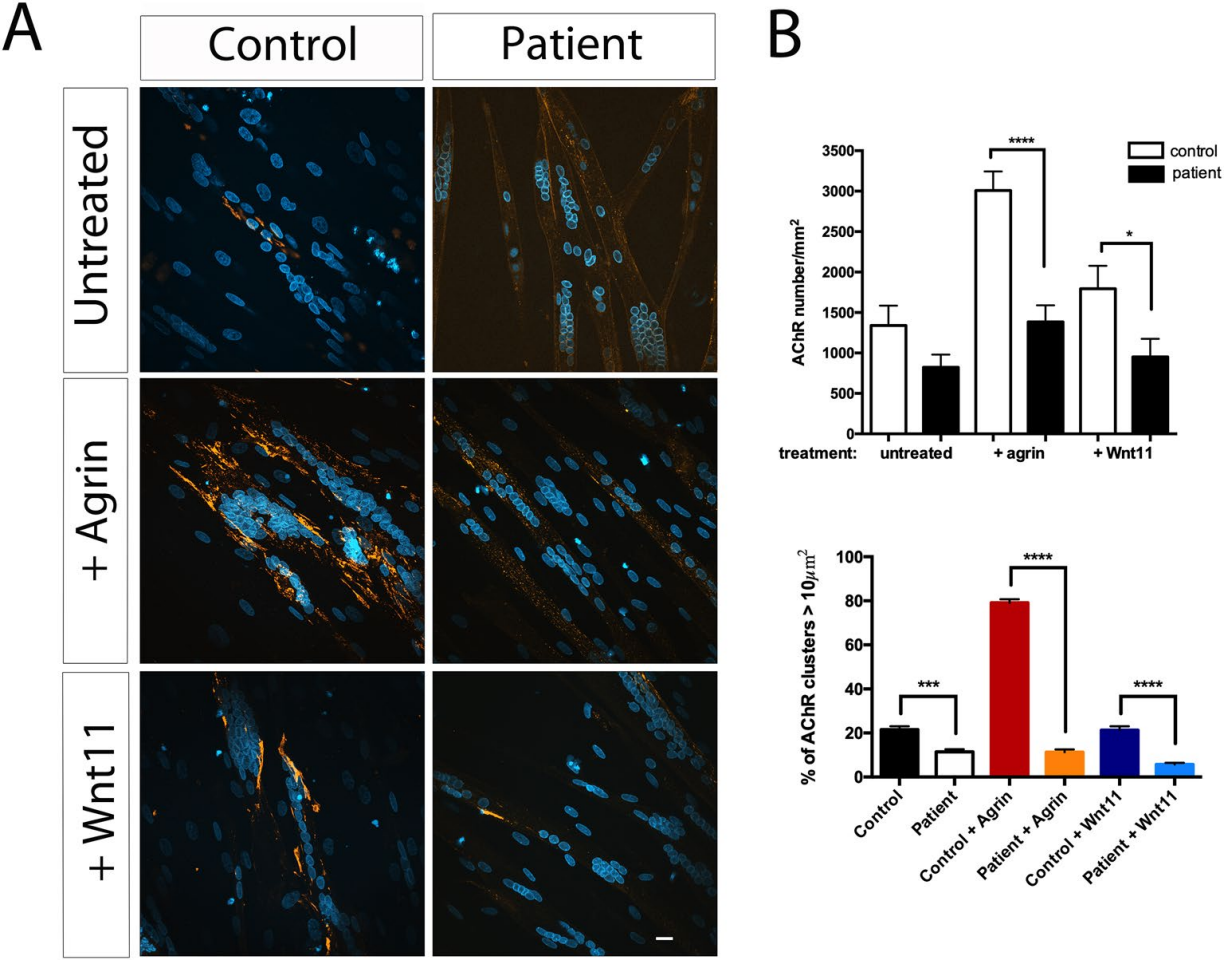
Neural agrin- and Wnt11-induced AChR aggregation in cultured patient myotubes. (**A**) Primary culture of human myotubes from the patient and healthy control differentiated into myotubes. After treatment or not with neural agrin (0.4 μg/ml) or Wnt11 (10 ng/ml) during 16 h, AChR aggregates and nucleus are labelled with α-BGT-TRITC and DAPI respectively and visualized with ApoTom microscope. At least, 70 myotubes were analyzed for each condition, Scale bar 10 µm. (**B**) Histogram of AChRs number per mm^2^ and percentage of AChR clusters > 10 μm^2^. No effect of the agrin or Wnt11 treatment in AChR clustering was observed on the patient's myotubes compared with the control. A decrease of AChR cluster size was observed in the patient’s myotubes compared with controls (*p < .05, **p < 0.01, ***p < 0.001 and ****p < 0.0001, Student’s test).

### Mutation analysis

After analysis of the NGS panel of CMS causing genes, Sanger sequencing revealed a novel homozygous c.1820A > G (p.Tyr607Cys) mutation in exon 14 of *LRP4* gene encoding the β1 propeller domain, important for the binding with neural agrin (Fig. 1D) and a list of non-pathogenic variants (Supplemental Table). The *LRP4* variant affects an evolutionary conserved nucleotide and amino acid, in a highly preserved region of the protein. No pathogenic variant was found in other CMS genes. The unaffected parents and brother could not be tested.

The *LRP4* p.Tyr607Cys residue is predicted as pathogenic by all prediction softwares (SIFT, Mutation Taster, Polyphen, Align-GVGD). Mutations in this domain have previously been reported to alter Wnt signaling and cause bone diseases, including CLS^1^.

### Expression studies

#### Mutation of the β1-propeller domain of LRP4 decreases neural agrin- and Wnt11-induced AChR aggregation in cultured patient myotubes

To evaluate the impact of the *LRP4* p.Tyr607Cys mutation on AChR aggregation, we studied primary muscle cells from a muscle biopsy of the patient. Cells were treated for 16 h with neural agrin (0.4 µg/ml) or Wnt11 (10 ng/ml) and then labelled with α-bungarotoxin-TRITC to detect AChR aggregates (Fig. 2A). A decrease of 85% with agrin and 55% with Wnt11 of aggregates was observed in patient-derived myotubes compared with control (Fig. 2A,B). Additionally, in both conditions, a decrease of AChR cluster size was observed in patient’s myotubes compared with the control (Fig. 2B). These results strongly suggest that the *LRP4* p.Tyr607Cys mutation disrupts agrin- as well as Wnt11-induced AChR clustering.

#### Mutation in the β1 propeller domain of LRP4 impairs the binding to neural agrin in vitro

**Figure 3:**
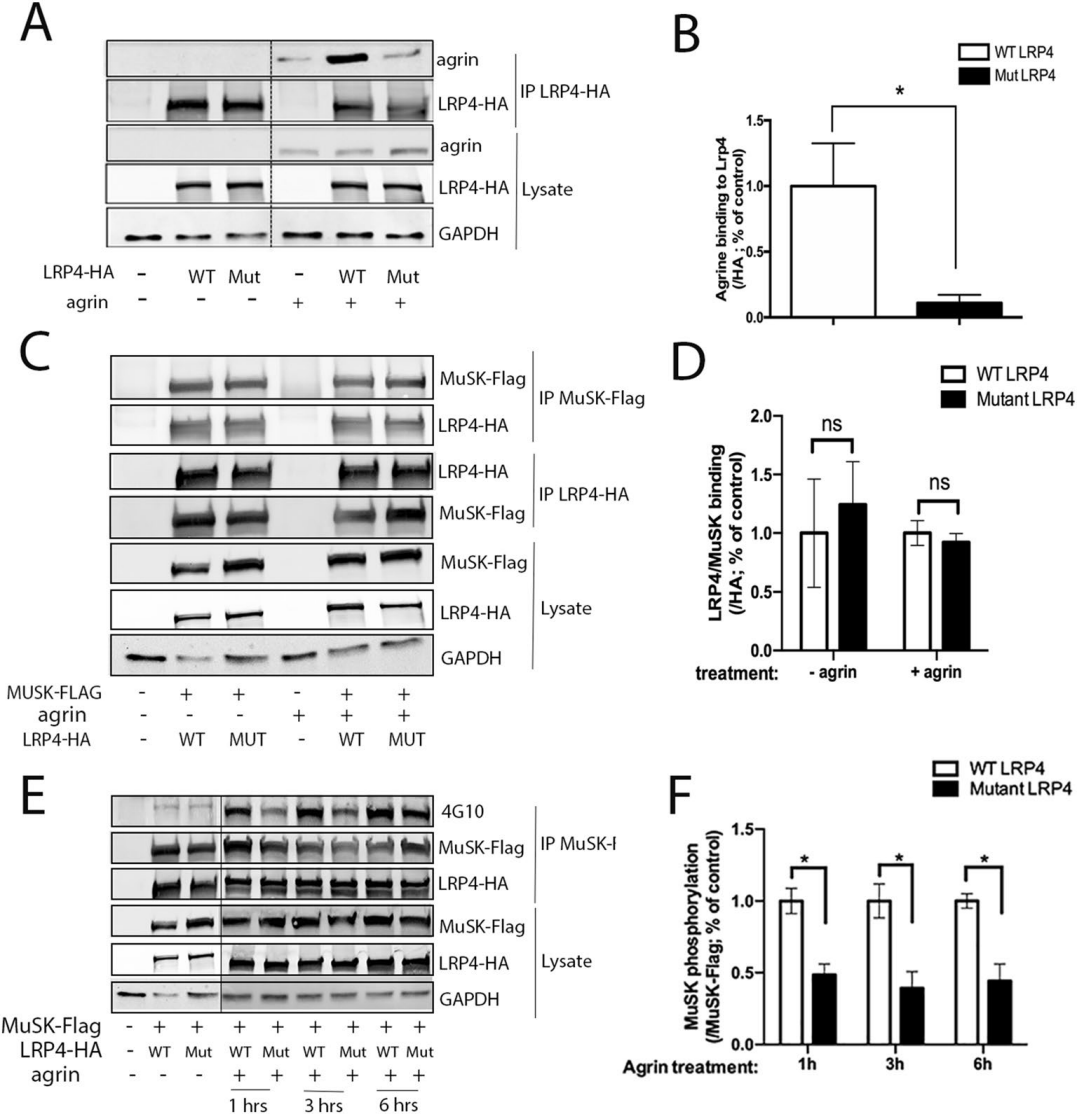
Alteration of agrin- mediated MuSK signaling induced by mutation in *LRP4.* (**A**) The LRP4 mutant inhibits agrin binding in vitro. HEK293T cells were transfected with wild-type (WT) or mutant (Mut) LRP4-HA plasmids. After immunoprecipitation of LRP4-HA and incubation with or without neural agrin (1 μg/ml), the level of agrin binding to LRP4 is followed by immunobloting with anti-agrin antibody. Lines indicate that intervening lanes have been spliced out. (**B**) Agrin binding to LRP4 shows a significant impairment of agrin binding to LRP4 mutated in vitro*.* (**C**) The LRP4 mutant does not affect its binding to its coreceptor MuSK. After co-expression of MuSK-Flag and LRP4-HA in HEK293T cells and immunoprecipitation of MuSK-Flag or LRP4-HA with anti-Flag and anti-HA respectively, the level of MuSK or LRP4 is followed by immunoblotting with anti-Flag or anti-HA antibodies. (**D**) LRP4 binding to MuSK doesn’t show significant changes of LRP4/MuSK binding when LRP4 is mutated in vitro. (**E**) The mutation in LRP4 inhibits agrin-mediated regulation of MuSK phosphorylation. HEK293T cells were transfected with MuSK-Flag and WT LRP4-HA or mutant LRP4-HA with or without agrin (0.4 μg/ml) for various times (1, 3 or 6 h). Phosphorylated MuSK was detected by immunoprecipitation of MuSK-Flag and followed by immunoblotting with anti-4G10 (MuSK phosphorylation), anti-Flag and anti-HA antibodies. Lines indicate that intervening lanes have been spliced out. (**F**) MuSK phosphorylation induced by agrin is significantly impaired when LRP4 is mutated in vitro. *p* < 0.05, Student’s test. N = 3 blots for each condition. Source data are provided as a Source Data file.

During NMJ formation, neural agrin, binds to the β1 propeller domain of LRP4 leading to MuSK phosphorylation and AChR clustering. To investigate the effect of *LRP4* p.Tyr607Cys mutation on this signaling pathway, we first assessed the binding between LRP4 and agrin. Control experiments show that wild-type and mutant LRP4 were similarly expressed and localized to the plasma membrane (Supplemental Fig. 1). Immunoprecipitated LRP4 proteins show a statistically significant 80% decrease in agrin binding to mutated LRP4 in vitro compared with control condition (*p* < 0.05, Student’s test) (Fig. 3A,B).

#### Mutation of the β1 propeller domain of LRP4 does not alter its binding to MuSK but impairs agrin-induced MuSK phosphorylation in vitro

LRP4 also interacts with MuSK with its β3 propeller domain. This interaction is critical for agrin-induced AChR clustering. We examined whether LRP4/MuSK binding is disrupted when the β1 propeller domain of LRP4 is mutated. A co-transfection and a co-immunoprecipitation of LRP4-HA (wild-type or mutant) and MuSK-Flag, respectively, showed no difference in LRP4/MuSK binding when the β1 propeller domain of LRP4 was mutated, compared with control (Fig. 3C). Quantification of the binding of LRP4 to MuSK is not statistically different (Fig. 3D), confirming that *LRP4* mutation does not affect LRP4/MuSK binding. We next evaluated MuSK phosphorylation in the presence of *LRP4* mutation. After different times of agrin treatment (1, 3 and 6 h) on HEK293T cells expressing both WT LRP4 or carrying the p.Tyr607Cys mutation and MuSK, we observed a significant 40% decrease of MuSK phosphorylation induced by agrin after one hour of incubation in the mutated condition (Fig. 3E,F).

#### Mutation of the β1 propeller domain of LRP4 inhibits its binding to Wnt11 in vitro

**Figure 4:**
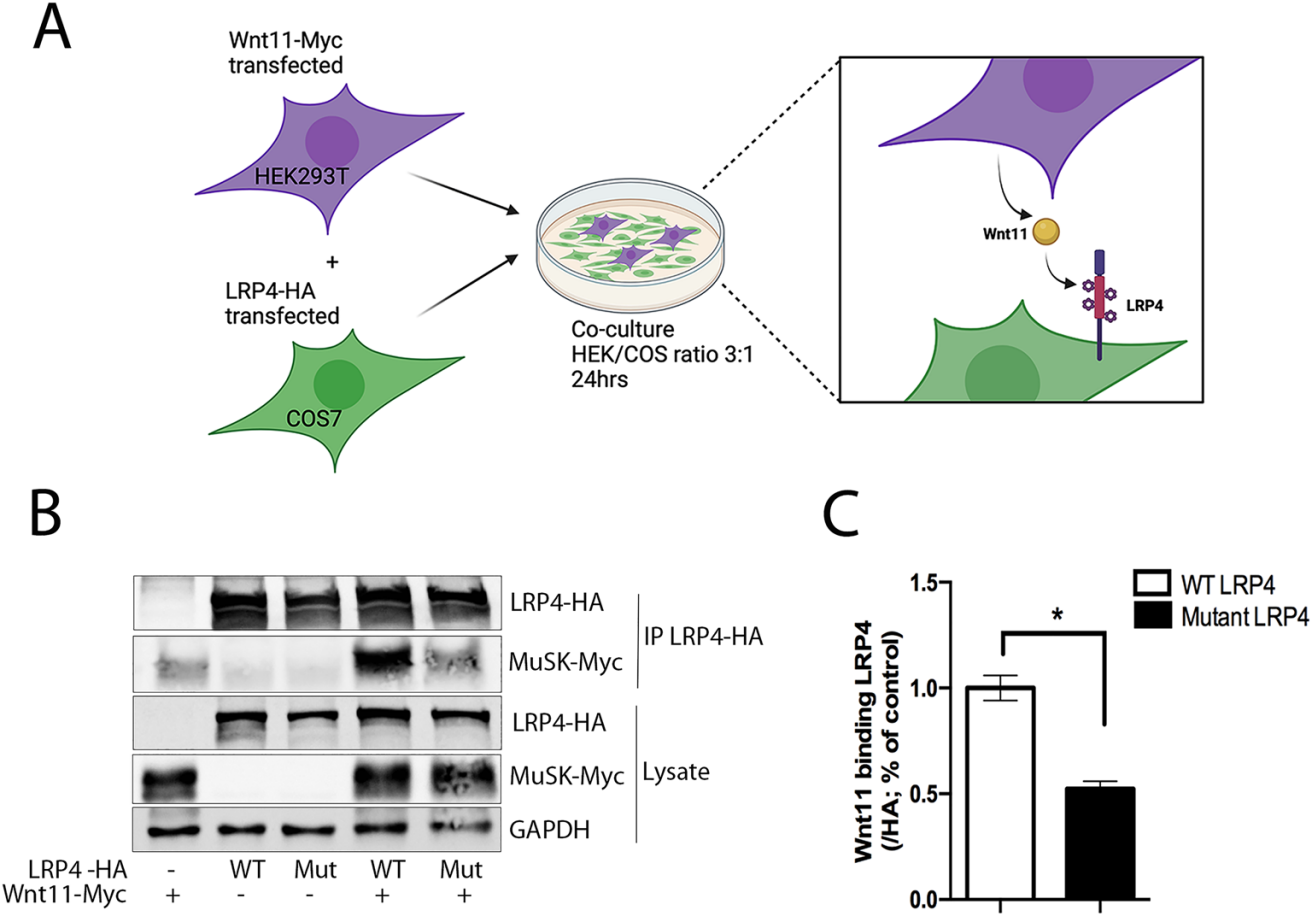
Mutation localized in the β1 propeller domain of LRP4 inhibits its binding to Wnt11 in vitro. (**A**) Wild-type (WT) or mutant (Mut) LRP4/Wnt11 co-culture and immunoprecipitation experiment. (**B**) Immunoprecipitation of LRP4-HA with Wnt11-Myc in COS7/HEK293T co-culture. Western blot using anti-HA and anti-Myc antibodies were realized on cell lysates in order to estimate the effect of the mutation in *LRP4* on its binding to Wnt11. (**C**) Wnt11 binding to mutated LRP4 shows a significant decrease compared with control. N = 3 co-culture and immunoprecipitation for each condition. *p* < 0.05, Student’s test. Source data are provided as a Source Data file. For A the figure was drawn by using BioRender.com.

A direct interaction between the extracellular domain of LRP4 and Wnt11 has been previously reported in vitro^15^. We assessed Wnt11 binding to the β1 propeller domain of LRP4 in the presence of p.Tyr607Cys mutation in a co-culture immunoprecipitation assay^16^. After 24 h of co-culture with LRP4 expressed on COS7 cells surface and Wnt11 secreted by HEK293T cells, immunoprecipitation of LRP4 showed direct interaction between the two partners (Fig. 4A). We observed a significant 45% decrease in Wnt11 binding to mutated LRP4 compared with LRP4 control (Fig. 4B). These results suggest that the binding of Wnt11 to the extracellular domain of LRP4 involves its β1 propeller domain and that the mutation alters this binding (Fig. 4C).

#### Three-dimensional structure modelling of the mutated β1 propeller domain of LRP4 suggests distal perturbations of agrin binding site

**Figure 5:**
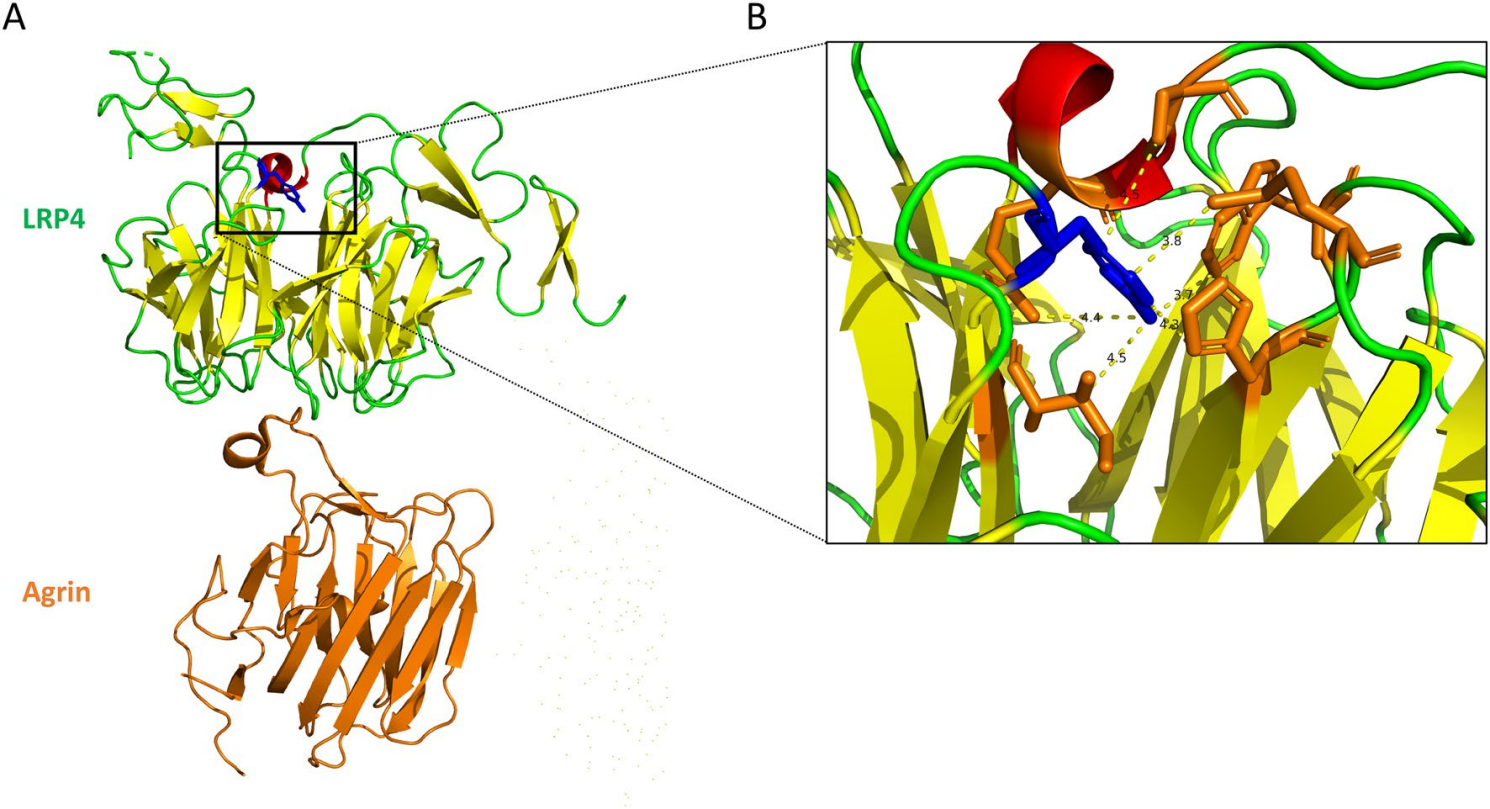
3D view of β1 propeller domain of LRP4 in complex with agrin involving Tyr607 mutation. (**A**) Backbone representation extracted from the crystal structure of LRP4, in complex with agrin (colored in orange), using PDB accession number 3V64. Tyr607 mutation is colored in blue. (**B**) Zoom view of the side chain interactions between Tyr607 (colored in blue) and neighbor residues (colored in orange). Inter side-chain distances lower than 5 Å are indicated by dash lines. For A and B figures were extracted from PyMol software.

In this study, we demonstrated that p.Tyr607Cys mutation in the β1 propeller domain of LRP4 affected its capacity to bind agrin and Wnt11 in vitro. The high-resolution structure of LRP4 in complex with agrin was previously resolved by X-ray diffraction showing that agrin–LRP4 forms a 2:2 tetrameric complex. By analyzing the atomic structure of this complex, we observed that *LRP4* mutation, at the interface with the flanking EGF modules, is not directly involved in the agrin-LRP4 binding (Fig. 5). Thus, *LRP4* p.Tyr607Cys mutation, by locally perturbing interactions of the β1 propeller domain, would alter the affinity for agrin without directly interacting with this partner. Furthermore, the binding site of Wnt11 on LRP4 is not yet well known but we cannot exclude that the Tyr607Cys mutation directly inhibits the formation of the LRP4/Wnt11 complex as demonstrated in vitro. Similarly, the structural analysis of two other mutations in the β1 propeller domain of LRP4 identified as responsible for Cenani–lenz syndrome (Asp529Asn, Arg545Trp), revealed that these exposed residues are also not directly involved in the agrin/LRP4 interaction, but such substitutions could affect the global stability of LRP4 and can consequently destabilize the agrin/LRP4 complex (data not shown).

## Discussion

Our work identifies a new homozygous missense mutation (c.1820A > G, p.Tyr607Cys) in the β1 propeller domain of LRP4 that binds to neural agrin and responsible for an atypical CMS case associated with CLS. Patient had clinically and electroneuromyographically demonstrated muscle weakness and fatigability associated with congenital malformations of the hands and feet and a horseshoe kidney, with no significant improvement of CMS symptoms with anticholinesterase treatments.

The majority of identified mutations in the *LRP4* gene cause bone disease including CLS and sclerosteosis 2 but the association of CMS and CLS has never been reported before in the same patient^1–5, 14^. CMS due to *LRP4* mutation are extremely rare with only three patients reported with variable phenotypes ranging from early and severe myasthenia to milder forms for one and two cases respectively^5, 14^. All these CMS patients carried missense mutations localized in the third β propeller domain of LRP4, with impairment of MuSK binding, activating and phosphorylation induced by agrin, without altering Wnt3a-suppressive activity in vitro^5, 14^. Our study highlights that a mutation in the β1 propeller domain of LRP4 alters its binding to agrin leading to neurotransmission defects whereas mutations described so far in this domain are exclusively reported to be responsible for CLS with limb and kidney malformations without neuromuscular defects^1, 4^. However, we cannot exclude that some patients with missense mutations localized in the β1-propeller domain of LRP4 may have altered neurotransmission that would need to be tested by ENMG. Consequently, differences between patients with LRP4 myasthenia described so far and our patient with both CMS and CLS could be due to the consequences of direct binding defects of agrin to the mutated β1 propeller domain of LRP4.

The binding of agrin released from the nerve terminal to LRP4 is a key mechanism in AChR clustering^9^. Several studies have also highlighted the role of secreted glycoproteins Wnt in the NMJ formation and more particularly in the AChR aggregation^15, 18^. Additionally, in vitro study showed that Wnt11 and Wnt9a, actor of canonical or non-canonical signaling, directly interact with the ecto-domain of LRP4^15^. Interestingly, our results from the patient’s primary myotubes show a significant decrease in the number and size of agrin- as well as Wnt11-induced AChR aggregates confirming that the LRP4 mutation localized in β1 propeller domain inhibits MuSK signaling acting on AChR aggregation at the postsynaptic membrane. Furthermore, our in vitro results confirm a disruption of agrin/LRP4/MuSK signaling when LRP4 is mutated, with a decrease in direct agrin binding leading to a defect of agrin-induced MuSK phosphorylation. Similarly, Wnt11-binding to mutated LRP4 is also decreased in vitro, results that corroborate the findings of Zhang et al. and indicate that Wnt11 binding to LRP4 is in part mediated by its β1 propeller domain^15^. Overall, these results demonstrate an important role for the β1 propeller domain of LRP4 in agrin/MuSK signaling leading to AChR aggregation at the postsynaptic membrane, which may explain the CMS phenotype observed in the patient. In addition, studies suggest that a ternary complex containing ColQ, perlecan and MuSK is required for AChE clustering, with MuSK playing an essential role in the synaptic localization of AChE at the NMJ^17, 19^. Previous investigations have also demonstrated that mutations in the β3 propeller domain of LRP4, responsible for congenital myasthenic syndrome, modified its binding and the function of its co-receptor MuSK, leading to AChE deficiency in the patient's biopsy^14^. In our study, although the mutated β1 propeller domain of LRP4 does not disturb LRP4/MuSK interaction, it does affect MuSK function, which could lead to impaired ColQ binding and therefore AChE deficiency in the patient's NMJ. However, this hypothesis could not be tested given the lack of NMJs and in vitro microelectrode studies in the muscle biopsy of the patient.

Here, we demonstrated that the in vitro ability of mutated LRP4 to bind agrin and Wnt11 was significantly reduced without altering the expression or cellular localization of LRP4 in a heterologous expression system. As revealed by the atomic structure of agrin/LRP4 complex, the side chain of highly conserved Tyr607 is not directly exposed at the surface of the β1 propeller domain but is mostly buried and involved in a network of interaction with side chains of Trp520, His562, Met564, Ile605, Arg694, Pro696, with inter-side chain distances lower than 5 Å. The substitution of the aromatic Tyr residue by a small Cys residue requires a local rearrangement of the neighbor residues to at least partially maintain the network of interaction between side-chains. Such distal modulation has been described as a general evolutionary approach to modulate the affinity of a given domain for its partners^20, 21^. *LRP4* p.Tyr607Cys mutation might only slightly affect the global stability and localization of LRP4 but induces local perturbations that propagate toward the binding sites of agrin, and consequently decreases their affinity^20, 21^. Moreover, as suggested by Zhang et al., we cannot exclude that the mutation Tyr607Cys directly inhibits the formation of the Wnt11/LRP4 complex even if the binding site of Wnt11 on LRP4 is not yet well known^15^. On the other hand, as observed for Tyr607 in this study, the mutated residues Asp529 and Arg545 on the surface of the β1 propeller domain of LRP4 identified as responsible for CLS, are not directly involved in the interaction with agrin but their substitutions may also affect the global stability of LRP4 and consequently its binding with its partners^1, 2^. These latest insights support the possibility of altered neurotransmission in CLS patients harboring a mutation in the β1 propeller domain of LRP4.

In addition to its major role in the AChR clustering, LRP4 also plays an important role in organogenesis^22^. Abnormal limb development in CLS indicates that LRP4 is a crucial player in the control of limb bud development in humans. The exact molecular mechanisms involved in this malformation have been studied and elucidated in the knockout mouse^23, 24^. Indeed, during limb development, the distalmost ectodermal region of the limb bud forms the so-called apical ectodermal ridge which is a very important signaling center. In *Lrp4* -/- mouse, this ridge is abnormal very early in development inducing abnormal pattern of expression of molecules like FGF8 (involved in proximo-distal growth of the limb), Sonic hedgehog (SHH; involved in antero-posterior patterning of the bud), BMP2 and 4 (implicated in the control of interdigital apoptosis) and Wnt7a (controlling ventro-dorsal polarity)^25, 26^. Taken together, these findings can then explain the observed human phenotypes such as brachydactyly due to growth impairment or syndactyly due to defect of interdigital apoptosis.

Furthermore, LRP4 is a regulatory protein of the Wnt signaling pathway by competing with LRP5 and LRP6 ligand-binding of importance for bone formation as well as limb and kidney development^26^. In CLS patients, several studies have hypothesized that loss of LRP4 during limb development could lead to an overactivation of LRP6 and in consequence causes an increase in Wnt signaling^1–3, 26^. Interestingly, we show that a mutation in the β1 propeller domain of LRP4 leads to a decrease in its binding to Wnt11 at the NMJ, whereas this same mutation is likely responsible for an increase in Wnt signaling involved in limb malformation. Additionally, studies of different models of LRP4-deficient mice also show that embryos exhibit both limb and kidney formation anomalies as well as major defects of NMJ formation^23, 24^. Finally, fully viable LRP4 hypomorphic phenotypes highlight the early effect of impaired or reduced LRP4 signaling on limb development, followed by a later involvement of synaptic abnormalities^23^. These latter results demonstrate that LRP4 domains differentially regulate limb development and synaptic plasticity that may explain the late CMS diagnosis in our patient.

In summary, we report the identification of a novel homozygous recessive mutation localized in the β1 propeller domain of LRP4 in a consanguineous family responsible for an atypical form of CMS with limb malformation. To date, mutations in the *LRP4* gene have been reported in several different phenotypes including CLS and CMS but never associated together in the same patient. Besides, this study appears useful for the future diagnosis and treatment of patients with co-existing CLS and CMS and highlights the importance of structural and functional studies in the diagnosis and possible treatment of these original forms.

## Methods

### Participants

Informed consent was obtained from all subjects and/or their legal guardians. The patient was referred to the French reference center for CMS for diagnosis and underwent standardized clinical and electrophysiological evaluations performed according to standardized protocols at the Pitié-Salpêtrière Hospital as already described^27^. Human muscle biopsies from the patient or healthy control were obtained from the BTR (Myobank-AFM, authorization AC-2019-3502) and myoblasts are cultivated as previously described^28–30^.

### Genetic analysis

Using patient genomic DNA isolated from the blood sample, the identification of variants was carried out on the basis of NGS-based screening of 54 genes involved in muscle excitability (list on request), among which 30 CMS causing genes, including *LRP4,* using a SeqCapEZ capture design (Nimblegen), and a MiSeq sequencer (Illumina). Variants were identified through a bioinformatics pipeline (Genodiag, Paris, France) and filtered according to their frequency in the general population (GnomAD) and in the patient’s sample. Copy number variations (CNVs) were searched for by a dedicated algorithm based on a comparison of the normalized number of reads of each region among the 12 samples of the sequence run. Primer design for amplifying *LRP4* gene was performed using Primer3 software. The patient genomic DNA was amplified by PCR and Sanger-sequenced. Reference transcript used for *LRP4* gene was NM_002334.3.

### Plasmids

We used plasmids containing full-length human *LRP4* cDNA, mouse *MuSK* cDNA and mouse *Wnt11* cDNA expressing C-terminal HA-tagged, Flag-tagged and Myc-tag respectively for co-culture and co-immunoprecipitation assay. Mutant *LRP4* plasmid carrying c.1820A > G (LRP4-Mut) was generated by GenScript Biotech (Chinese) by directed mutagenesis.

### Cell culture and transient transfection

HEK293T and COS7 cells (ATCC) are cultured at 37 °C and 5% CO2 in a DMEM medium (Dulbecco's modified Eagle's medium—Thermo Fisher scientific) containing 10% fetal calf serum (FCS) with 1% penicillin–streptomycin (Thermo Fisher scientific), and transfected with FuGENE6 transfection reagent (Roche). The co-culture co-immunoprecipitation assay involves the transfection of two potential interactors in two different cell types, followed by co-culture and immunoprecipitation assay, providing a simple and reliable method to detect specific interactions as previously described^16^.

A primary culture of human myoblasts from patient or healthy control (25 years old) was carried out by the human muscle cell immortalization platform (Myoline facility). Cells were isolated, purified and differentiated into myotube, as previously described^28^. After recombinant rat agrin (R&D systems, 0,4 μg/ml) and Wnt11 (R&D systems, 10 ng/ml) treatment for 16 h, myotubes were labelled with Tetramethylrhodamine α-bungarotoxin (α-BGT-TRITC, Thermo Scientific, 1:500) and visualized with ApoTom microscope (ApoTom.2, Zeiss) equipped with a camera and a 40X digital oil-immersion objective, as previously described^27^. The same settings were used to compare control and mutant myotubes. The number and area of AChR clusters were quantified with ImageJ (version 2.0.0) using a standardized method based on thresholding the intensity of the objects of interest. Each experiment was done in triplicate.

### Agrin-Lrp4 interaction in vitro

After transfection, LRP4-HA (wild-type or mutated) was immobilized with beads using anti-HA antibody (Ozyme, 1:100) and incubated with recombinant agrin (1 µg/mL), as previously described^16^. Interactions with LRP4 and agrin were determined by immunoprecipitation and western blot using an anti-agrin antibody (Millipore, 1:1000).

### MuSK interaction and phosphorylation

To evaluate whether the LRP4 mutation disrupts both the interaction with its co-receptor MuSK and time-dependent agrin-induced phosphorylation of MuSK, HEK293T cells were co-transfected 24 h with *MuSK* plasmid and wild-type or mutant *LRP4* plasmid (2 µg of each plasmids) and treated when necessary with recombinant agrin (0.4 µg/ml, 1, 3 and 6  h). A co-immunoprecipitation of LRP4-HA and MuSK-Flag using an anti-HA (Ozyme, 1:100) or anti-Flag (Ozyme, 1/150) was performed and then revealed by Western blotting. MuSK phosphorylation and tagged MuSK transfected were revealed by an anti-phospho-tyrosine antibody (Millipore, 4G10, 1:1000) and an anti-Flag antibody (Ozyme,1:5000) respectively.

### Western blotting

Total or immunoprecipitated proteins, from single transfection (LRP4 plasmids), co-transfection (LRP4 and MuSK plasmids) or co-culture (LRP4 and Wnt11 plasmids), were separated on 10% SDS–polyacrylamide gel before being transferred to a nitrocellulose or PVDF membrane as described^16^. The primary antibodies were anti-4G10 (Millipore, 1:1000), anti-agrin (Millipore, 1:1000), anti-GAPDH (Abcam, 1:10,000) and anti-transferrin (Invitrogen, 1:500). The fluorescent secondary antibodies used with ChemiDoc MP machine (Bio-Rad) were Star-Bright goat anti-mouse and Star-Bright goat anti-rabbit (Bio-Rad, 1:5000). GAPDH was used as a loading indicator for biotinylated proteins and lysates respectively.

### Biotinylation assay

After 24 h of transfection of wild-type or mutated LRP4 plasmids into HEK293T cells, cell surface proteins were isolated by biotinylation and bound proteins were precipitated with streptavidin beads as previously described^5^.

### Three-dimentional modeling

The crystal structure of agrin and hLRP4 (PDZ accession number 3V64) was analyzed using PyMol software (Schrodinger, LLC, version 2.4.0a0) and the crystallographic model was generated by PyMol^9^.

### Statistical analysis

Statistical analysis and histograms were generated using GraphPad statmate software (Prism6) and statistical results are presented as follows: ns = no significant (*p* > 0.05), *p < 0.05, **p < 0.01, ***p < 0.001 and ****p < 0.0001. In all figures, the data are presented as mean ± SEM.

### Ethical approval

This human study was conducted in accordance with the ethical standards defined in the 1964 Declaration of Helsinki and its subsequent amendments. The study was approved by national ethic committees (DC-2012-1535 and AC-2012-1536) of Institute of Myology.

### Informed consent

Consent for publication has been obtained from the patient.

### Supplementary Information

Supplementary Figures.

Supplementary Information.

Supplementary Table 1.

## Data Availability

Anonymized data not published in this article will be made available by request from the corresponding author. Publications cited in the manuscript are available online, as referenced in the reference section. All data generated or analyzed during this study are included in this published article (and its supplementary information files) or are available from the corresponding author on reasonable request.

## Abbreviations

LRP4: LDL receptor related protein 4
CMS: Congenital myasthenic syndrome
NMJ: Neuromuscular junction
CLS: Cenani–Lenz syndrome
AChR: Acetylcholine receptor
MuSK: Muscle specific kinase
CMAP: Compound muscle action potential
RNS: Repetitive nerve stimulation
ENMG: Electroneuromyography
