# Supplementary material for: New mutation in the β1 propeller domain of LRP4 responsible for congenital myasthenic syndrome associated with Cenani–Lenz syndrome
Source: Sci Rep. 2023 Aug 28;13:14054. doi: 10.1038/s41598-023-41008-5 (PMC10462681; doi:10.1038/s41598-023-41008-5)
Supplement: Supplementary file 1 — Supplementary Figures. [file 41598_2023_41008_MOESM1_ESM.pdf]

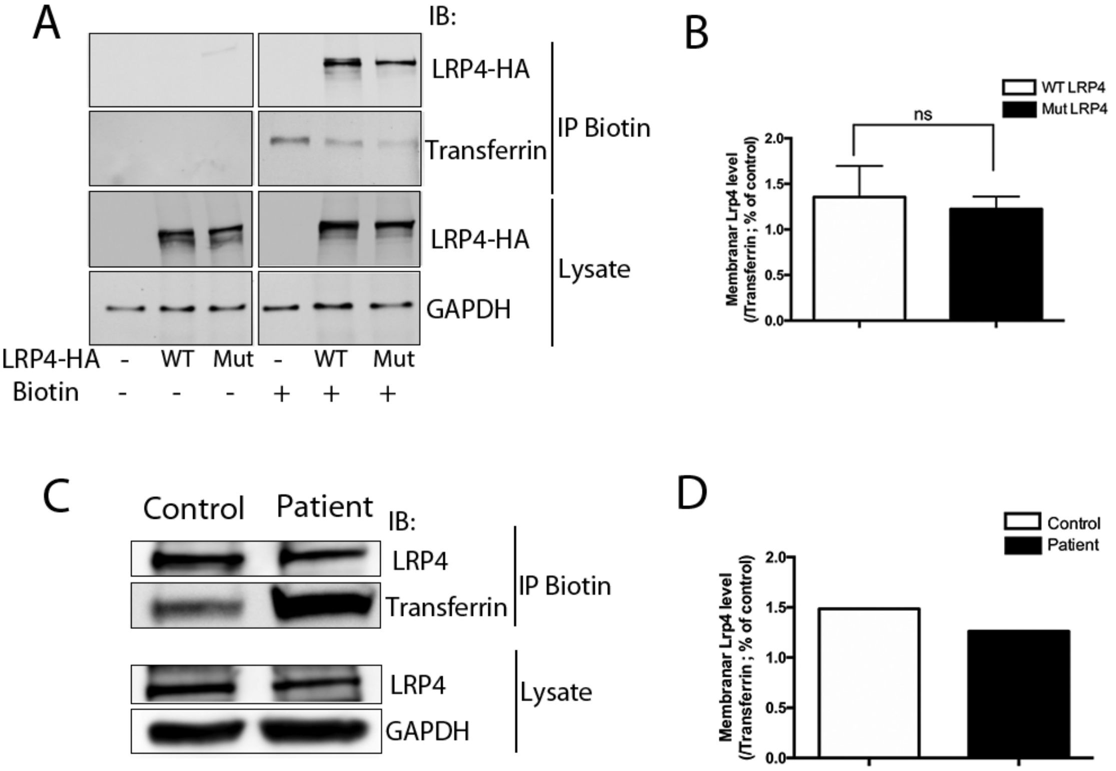

Supplemental Figure 1: Mutation in *LRP4* does not disrupt its expression and plasma membrane addressing in vitro and ex vivo. (A) Western blotting detection of membrane-bound LRP4 and total LRP4 in HEK293T cells. Membrane proteins were biotinylated and immunoprecipitated with streptavidin. Transferrin serves as an expression loading control for membrane proteins and GAPDH serves as a total protein loading control. N=3 biotinylated and immunoprecipitation experiment for each condition. (B) LRP4 wild-type (WT) and mutant (Mut) are similarly expressed and addressed on the cell surface of HEK293T (N=3, p>.05, Student's test). (C) Western blotting detection of membrane-bound LRP4 and total LRP4 in cultured human myotubes. Membrane proteins were biotinylated and immunoprecipitated with streptavidin. Transferrin serves as an expression loading control for membrane proteins and GAPDH serves as a total protein loading control. N=1 biotinylation experiment for each condition. (D) Patient and control LRP4 are similarly expressed and addressed on the cell surface of myotube. Source data are provided as a Source Data file.

H&E ATPase

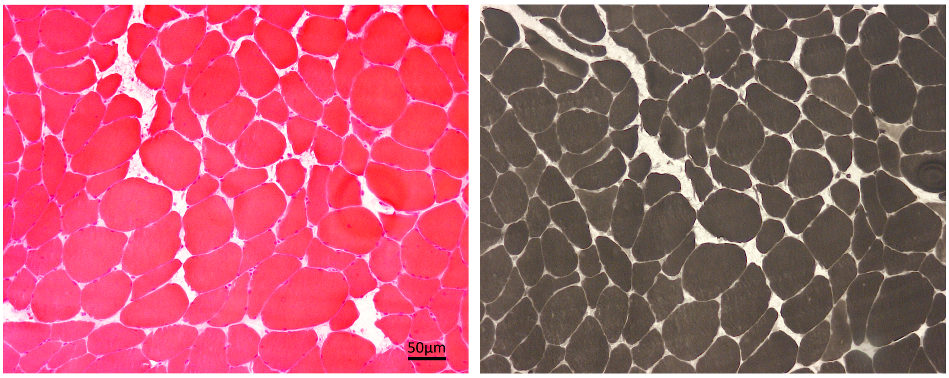

Supplemental Figure 2: Muscle histology on the patient's biopsy. Histological and histochemical analysis of the patient's muscle sections revealed fiber size variability on H&E staining and a predominance of type I myofibers on ATPase staining (pH 9.4).
