# Supplementary material for: New mutation in the β1 propeller domain of LRP4 responsible for congenital myasthenic syndrome associated with Cenani–Lenz syndrome
Source: Sci Rep. 2023 Aug 28;13:14054. doi: 10.1038/s41598-023-41008-5 (PMC10462681; doi:10.1038/s41598-023-41008-5)

Figure 3: LRP4/agrin binding in vitro

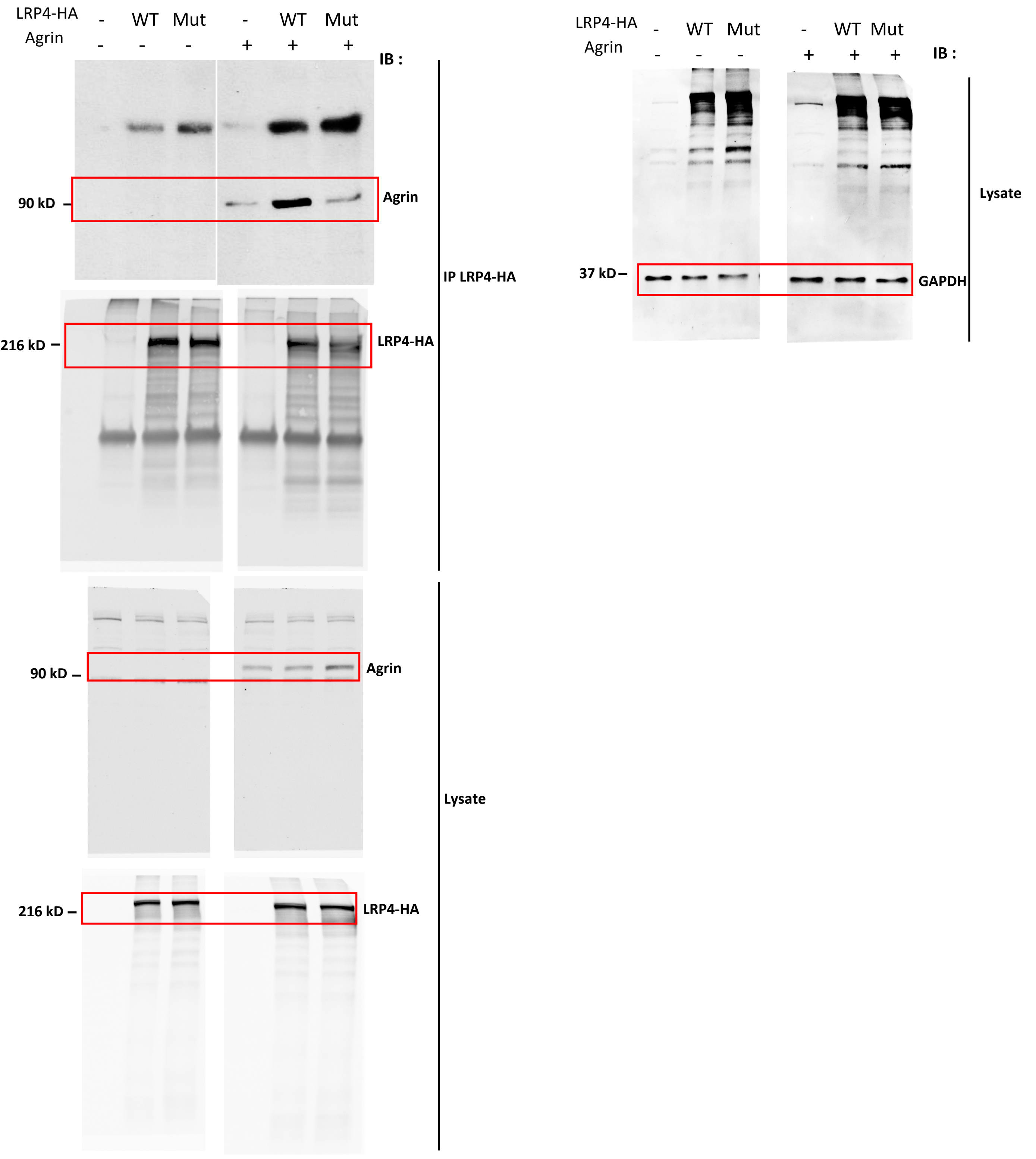

Figure 3: LRP4/MuSK binding in vitro

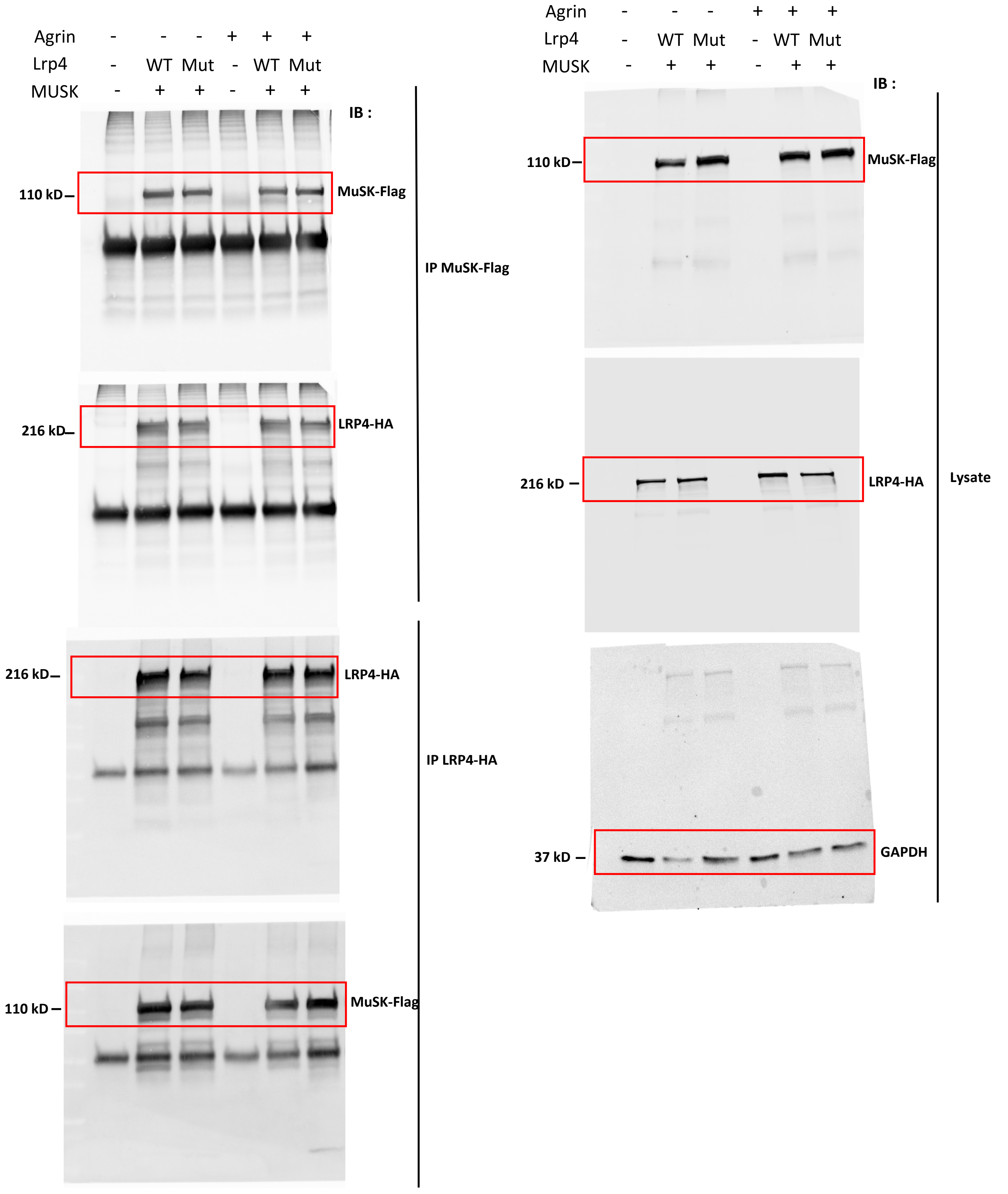

Figure 3: MuSK phosphorylation in vitro

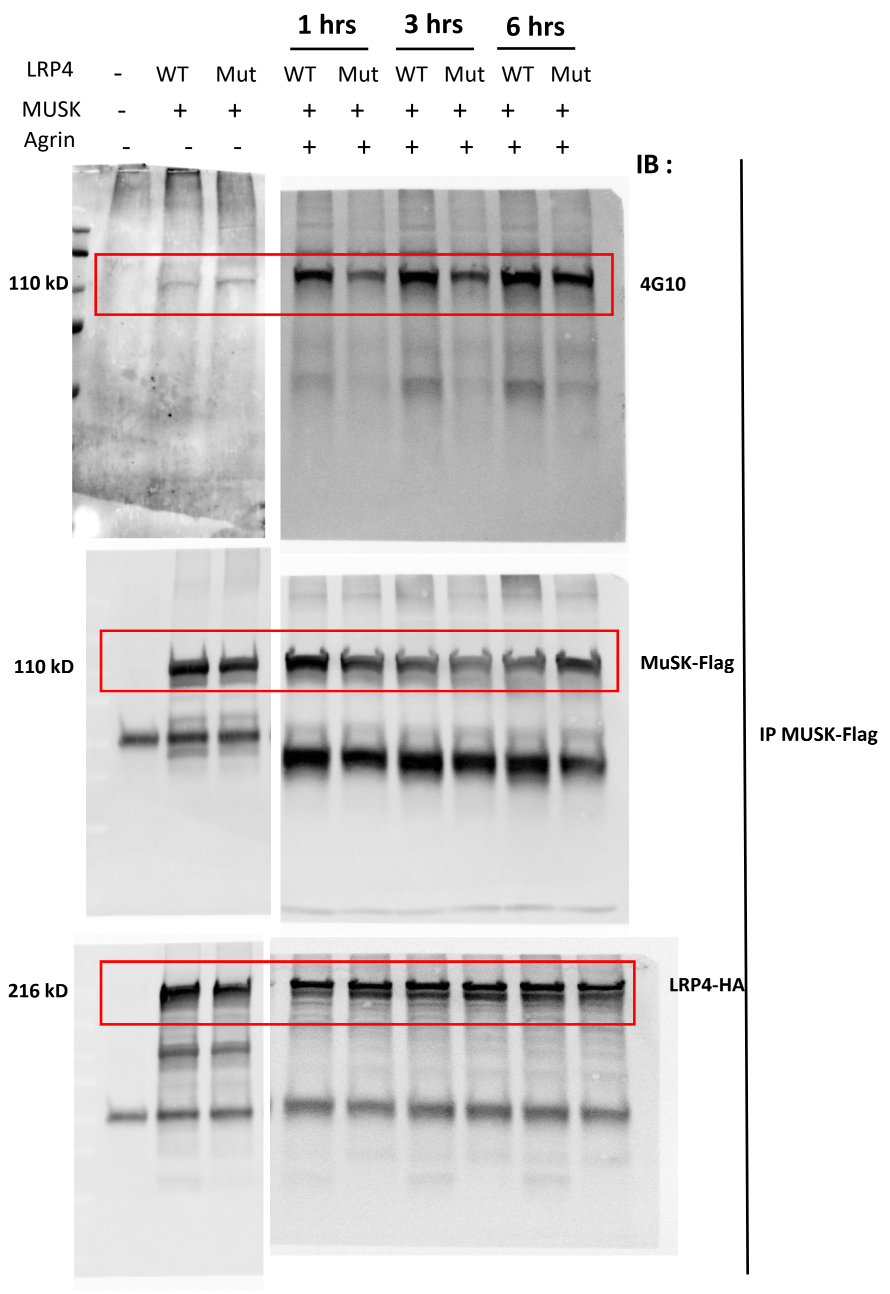

Figure 3: MuSK phosphorylation in vitro

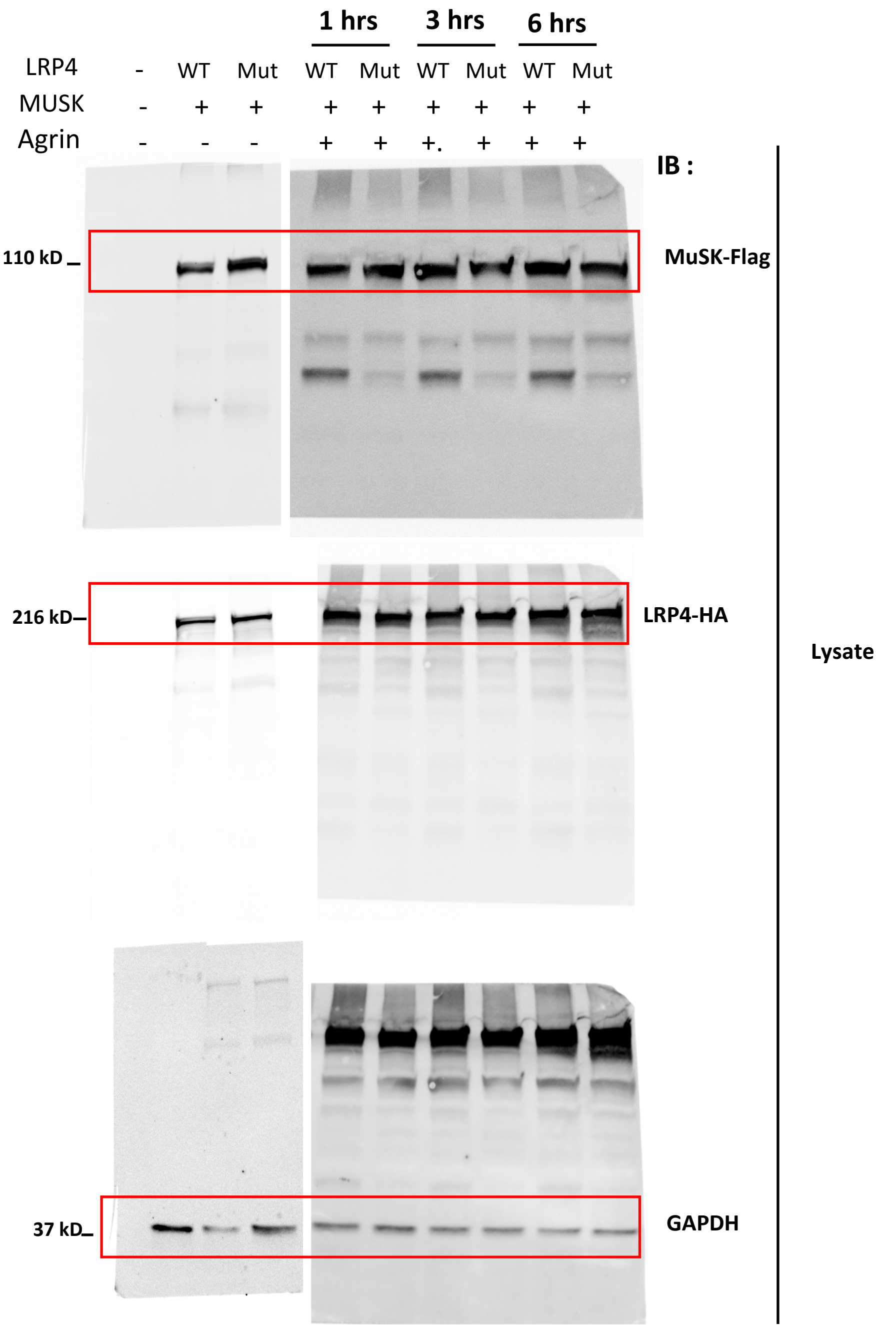

Figure 4: LRP4/Wnt11 binding in vitro

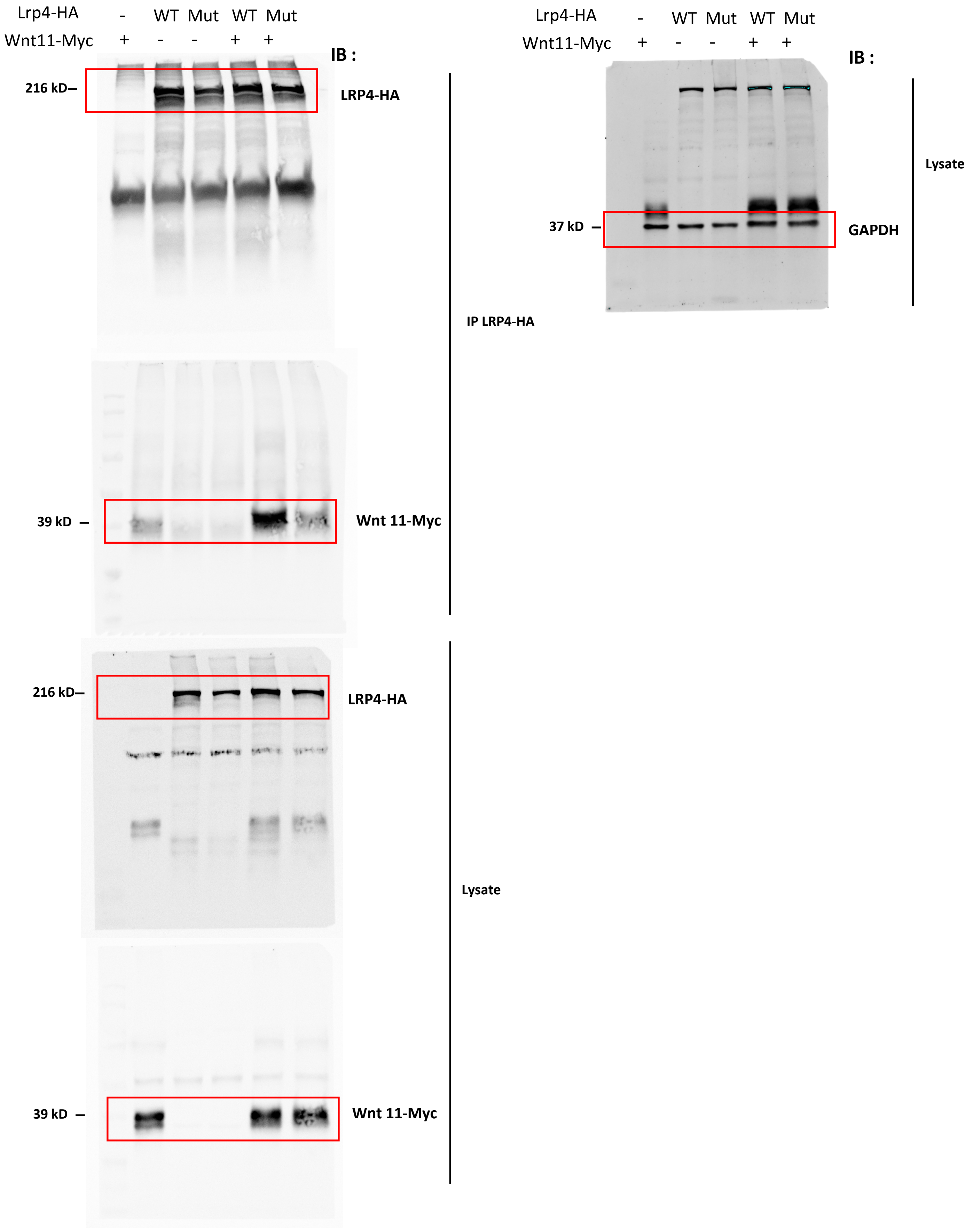

Supplementral Figure 1: LRP4 addressing *in vitro* and *ex vivo*

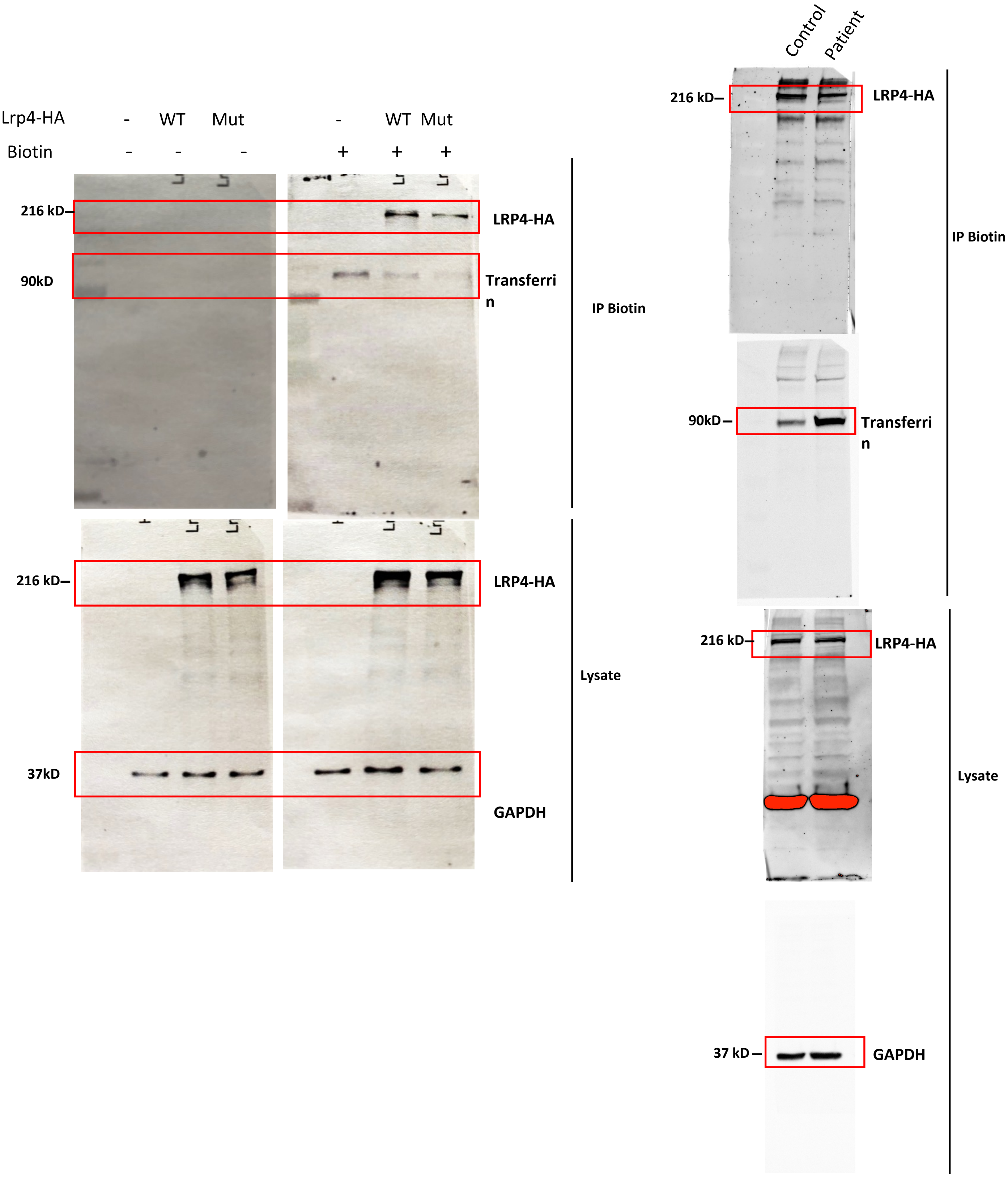

Supplement: Supplementary file 2 — Supplementary Information. [file 41598_2023_41008_MOESM2_ESM.pdf]
