# Supplementary material for: New mutation in the β1 propeller domain of LRP4 responsible for congenital myasthenic syndrome associated with Cenani–Lenz syndrome
Source: Sci Rep. 2023 Aug 28;13:14054. doi: 10.1038/s41598-023-41008-5 (PMC10462681; doi:10.1038/s41598-023-41008-5)
Supplement: Supplementary file 3 — Supplementary Table 1. [file 41598_2023_41008_MOESM3_ESM.pdf]

Gene name	HGVSc	HGVSp	zygosity
AGRN	c.3066A>G	p.Ser1022Ser	Homozygous
AGRN	c.3558T>C	p.Phe1186Phe	Homozygous
AGRN	c.4161T>C	p.Thr1387Thr	Homozygous
AGRN	c.6057C>T	p.Asp2019Asp	Homozygous
LAMA5	c.9235C>T	p.Arg3079Trp	Homozygous
LAMA5	c.7386T>C	p.Asp2462Asp	Homozygous
LAMA5	c.5698G>A	p.Val1900Met	Homozygous
LAMA5	c.5420T>C	p.Phe1807Ser	Heterozygous
MYO9A	c.3578G>A	p.Gly1193Glu	Heterozygous
CHAT	c.1135G>A	p.Val379Met	Homozygous
CHAT	c.1381G>A	p.Val461Met	Homozygous
CHAT	c.1395T>C	p.His465His	Heterozygous
CHAT	c.1641T>C	p.His547His	Heterozygous
SLC18A3	c.1559C>A	p.Ala520Glu	Homozygous
UNC13A	c.3101T>C	p.Leu1034Pro	Homozygous
CHRNB1	c.95A>G	p.Glu32Gly	Heterozygous
CHRND	c.12A>G	p.Pro4Pro	Homozygous
DOK7	c.220T>C	p.Leu74Leu	Homozygous
DOK7	c.887A>G	p.Gln296Arg	Heterozygous
DOK7	c.1113A>C	p.Ser371Ser	Heterozygous
DOK7	c.1134G>A	p.Ala378Ala	Heterozygous
DOK7	c.1185C>T	p.Tyr395Tyr	Heterozygous
LRP4	**c.1820A>G**	**p.Tyr607Cys**	**Homozygous**
LRP4	c.4937G>A	p.Arg1646Gln	Homozygous
LRP4	c.4660A>G	p.Ser1554Gly	Homozygous
LRP4	c.3256A>G	p.Ile1086Val	Homozygous
LRP4	c.1820A>G	p.Tyr607Cys	Homozygous
GMPPB	c.551A>G	p.Gln184Arg	Homozygous
ALG2	c.475A>G	p.Ile159Val	Heterozygous
SCN4A	c.4126A>G	p.Asn1376Asp	Heterozygous
SCN4A	c.1570A>G	p.Ser524Gly	Homozygous

Supplemental Table: Pathogenic and non-pathogenic variants identified in CMS related genes from the patient's genome. Next-Generation-Sequencing (NGS)-based screening of 30 CMS causing genes, including *LRP4,* was performed on the patient's genome. The identified pathogenic *LRP4* mutation described in this study (in bold) and non-pathogenic variants listed in this table. HGVSc: Human Genom Variation Society Coding sequence; HGVSp: Human Genom Variation Society Protein sequence.
